# Management of glioblastoma in elderly patients: A review of the literature

**DOI:** 10.1016/j.ctro.2024.100761

**Published:** 2024-03-10

**Authors:** Nektarios K. Mazarakis, Stephen D. Robinson, Priyank Sinha, Christos Koutsarnakis, Spyridon Komaitis, George Stranjalis, Susan C. Short, Paul Chumas, Georgios Giamas

**Affiliations:** a Royal Sussex County Hospital, University Hospitals Sussex NHS Foundation Trust, Eastern Rd, Brighton BN2 5BE, UK; b School of Medicine RCSI, Royal College of Surgeons in Ireland, 123 St. Stephen’s Green, Dublin 2, Ireland; c Department of Biochemistry and Biomedicine, School of Life Sciences, University of Sussex, Brighton BN1 9QG, UK; d Department of Neurosurgery, Leeds General Infirmary, Great George Street, LS1 3EX, UK; e Department of Neurosurgery, Evaggelismos Hospital, Ipsilantou 45-47, Athens, Greece; f Leeds Institute of Medical Research at St James’s Wellcome Trust Brenner Building St James’s University Hospital Leeds, LS9 7TF, UK

**Keywords:** Glioblastoma, Elderly, Surgery, Chemotherapy, Radiotherapy, Glioma

## Abstract

High grade gliomas are the most common primary aggressive brain tumours with a very poor prognosis and a median survival of less than 2 years. The standard management protocol of newly diagnosed glioblastoma patients involves surgery followed by radiotherapy, chemotherapy in the form of temozolomide and further adjuvant temozolomide. The recent advances in molecular profiling of high-grade gliomas have further enhanced our understanding of the disease. Although the management of glioblastoma is standardised in newly diagnosed adult patients there is a lot of debate regarding the best treatment approach for the newly diagnosed elderly glioblastoma patients.

In this review article we attempt to summarise the findings regarding surgery, radiotherapy, chemotherapy, and their combination in order to offer the best possible management modality for this group of patients. Elderly patients 65–70 with an excellent functional level could be considered as candidates for the standards treatment consisting of surgery, standard radiotherapy with concomitant and adjuvant temozolomide. Similarly, elderly patients above 70 with good functional status could receive the above with the exception of receiving a shorter course of radiotherapy instead of standard. In elderly GBM patients with poorer functional status and MGMT promoter methylation temozolomide chemotherapy can be considered. For elderly patients who cannot tolerate chemotherapy, hypofractionated radiotherapy is an option.

In contrast to the younger adult patients, it seems that a careful individualised approach is a key element in deciding the best treatment options for this group of patients.

## Highlights

• Glioblastoma management in the elderly is complex.
• Lack of clear definitions and randomised trials creates barriers to standardisation.
• Personalisation of treatment is required to balance survival with toxicity.

## Introduction

Glioblastoma (GBM) is a highly aggressive and infiltrative brain tumour and despite substantial advancements in understanding tumour biology, the outcome of untreated GBM remains poor with a median survival of 6.1 months and only 3.2 months in patients > 70 [1].

Following the landmark publication by Stupp et al, the clinical management of GBM was standardised [2]. However, only patients < 70 were eligible and subgroup analysis showed that the treatment was less effective with increasing age (hazard ratio (HR) 0.63 for patients 50–60 (p < 0.05), 0.72 for patients 60–65 (p = 0.096) and 0.8 for patients 65–71 (p = 0.340)) [3].

Several reasons have been advanced to explain the poor prognosis observed in elderly GBM patients, including reduced treatment intensity, poor response, increased toxicity, polypharmacy leading to drug interactions, and altered tumour biology [4], [5], [6], [7], [8], [9]. Additionally, elderly patients may more readily refuse treatment [8]. Consequently, there is no standard of care for elderly GBM patients and treatment varies between surgeons, units, regions, and countries. Treatment varies from palliation with no anti-cancer treatment to aggressive surgery followed by chemoradiotherapy [4], [7], [9].

Given this variation, there are calls for more research especially considering that the median age at diagnosis is 64 years [10]. However, there are numerous hurdles in promoting further research in management of GBM in the elderly population. Firstly, there is no clear definition of “elderly” with the cut off varying from > 60 to > 75 [6]. Additionally, population-based cancer registry studies have identified that elderly GBM patients are less likely to receive multi modal treatment. Surveillance, Epidemiology, and End Results (SEER) cancer registry data of 4,137 GBM patients ≥ 65 diagnosed 1994–2002 demonstrated a median overall survival (OS) of 4 months, and whilst 61 % of the patients underwent surgical resection, and 65 % received radiotherapy, only 10 % received chemotherapy within 3 months of their diagnosis [8]. Data from another SEER registry study of 2,836 GBM patients > 70 diagnosed 1993–2005 showed that only 46 % of the patients received both surgery and radiotherapy whereas 14 % of the patients received best supportive care [11]. Similarly, Swiss registry data revealed that 82 % of patients < 65 received surgery and/or radiotherapy whereas only 47 % of patients > 65 received surgery and/or radiotherapy. For patients > 75, only 25 % received with surgery and/or radiotherapy and 75 % received palliative care only [12]. Also, due to perceived poor prognosis and potential toxicity concerns elderly GBM patients are usually excluded from clinical trials limiting the available evidence to guide their treatment.

With improvement in healthcare, life expectancy is gradually increasing. Currently half of GBM patients are > 65, whilst 25 % are > 70 [5], [6]. However, given limited life expectancy in elderly GBM patients, management can be challenging, and the goal of treatment must balance increasing survival with maintaining a good performance status (PS) and limiting toxicity [9]. A Canadian population-based study identified that the percentage of patients (60–69, 70–79 and **≥** 80) who spent at least 50 % of their time as in-patient post GBM diagnosis was 45 %, 59 % and 76 % respectively [13]. Similarly, a retrospective cohort study of 5,029 GBM patients **≥** 65, showed that 21 % of the patients were hospitalized for at least 30 cumulative days between diagnosis and death and 22 % of all patients spent at least 25 % of their remaining lives as inpatients [14].

In this paper, we have reviewed various prognostic factors and treatment modalities used in the care of elderly patients with GBM with special reference to the landmark paper by Perry et al published in 2017 [15].

### Age

Age has been recognised as the most important prognostic factor for survival in GBM patients since the classic Radiation Therapy Oncology Group (RTOG) paper by Curran et al. Studying 1,578 patients from 3 trials they demonstrated that age > 50 was the most important factor associated with poor prognosis in GBM [16]. Further data from the Central Brain Tumor Registry of the United States, including patients diagnosed 1995–2009, showed that 2-year survival rate for patient aged 45–54, 55–64, 65–74 and **≥** 75 was 20.6 %, 14.2 %, 6.9 %, and 2.6 % respectively [10]. Updated data, including patients diagnosed 2009–2013, showed that 1-year survival rate for GBM patients aged 65–74 and **≥** 75 was 28.7 % and 12.1 % respectively, whilst the 5-year survival was 2.4 % and 1.1 % respectively [17].

### Performance status

Performance status also plays a major role in deciding patient suitability to receive aggressive multimodality treatment. Karnofsky performance status (KPS) > 70 is usually considered as cut off to decide eligibility for treatment and trial eligibility [7], [18]. However, KPS does not purely reflect a patients’ general status as often the devastating effects of the tumour itself can lead to neurological deficit and poor PS [5]. A recursive partitioning analysis investigating prognostic factors for GBM patients > 70, devised four prognostic subgroups according to age (>/<75.5), PS (KPS >/〈7 0) and type of surgery [19]. In addition, the presence of co-morbidities and the location of the tumour must also be considered to formulate appropriate treatment plans.

### Surgery

Surgery is important both to achieve a molecular integrated diagnosis and for cytoreduction. Several studies have investigated the effect of extent of resection (EOR) on survival and most have shown that greater resection results in improved OS [20]. However, elderly patients have higher risks of surgery related morbidity and mortality including the risks of general anaesthesia.

In a study combining three RTOG clinical trials (645 GBM patients), 19 % of the patients underwent total resection, 64 % partial resection and 17 % biopsy only. Median survival was 11.3, 10.4 and 6.6 months respectively [21]. Interestingly, 37 % of patients were 60–74, however patients > 75 were not included [21]. Similarly, a retrospective review analysed 128 GBM patients > 65 (range 65–83) in whom 88 patients had undergone biopsy and 40 had resection. Both groups had approximately similar median age and KPS. Median survival was 15.4 weeks in biopsy patients compared to 27 weeks in resection patients. The authors concluded that resection is associated with modest increase in survival in patients > 65 [22].

In a retrospective review of 76 patients ≤ 65, Martinez et al showed that gross total resection (GTR) was associated with longer survival compared to subtotal resection (STR) (p = 0.003) and biopsy (p = 0.004). The authors also showed in 62 patients > 65, GTR was associated with longer survival compared to STR (p = 0.001) and biopsy (p = 0.05) [23]. Similarly, a retrospective review of 345 GBM patients > 60 showed that EOR was an independent prognostic factor [24]. A further retrospective review of 394 GBM patient ≥ 65, demonstrated that on multivariate analysis surgical resection was an independent predictor of survival with GTR associated with 60 % reduction in risk of death compared to STR [25].

Chaichana et al studied 205 patients who underwent either biopsy or resection and matched 40 patients from each group for age, KPS, eloquent location and receipt of adjuvant treatment. They demonstrated that patients receiving surgery had a median survival of 5.7 months compared to 4 months in biopsy patients (p = 0.02). For patients ≥ 70, median survival in resection and biopsy groups was 4.5 and 3.0 months respectively (p = 0.03). The authors concluded that elderly patients with GBM can tolerate aggressive surgery with acceptable morbidity and mortality leading to better OS compared to patients undergoing biopsy [26]. In another retrospective review of 129 patients > 65 who underwent resection, Chaichana et al identified 6 risk factors associated with poorer outcome (KPS, chronic obstructive pulmonary disease, tumour > 4 cm in size, motor deficit, language deficit and cognitive deficit). The authors proposed using these prognostic markers in decision making for patients > 65 as patients with 0–1, 2–3 and 4–6 risk factors had median survival of 9.2, 5.5 and 4.4 months respectively [27]. One of the key messages was that age was not an independent factor in survival and other parameters such as neurological deficit, size of tumour and medical comorbidities might be more important.

Several further studies have shown that EOR correlates with OS. In a retrospective review of 103 GBM patients > 65, Ewelt et al showed that patients with GTR, STR and biopsy had a median survival of 13.9, 7 and 2.2 months respectively. In further analysis, they showed that surgery alone (resection or biopsy) has a median survival of 2.2 months, surgery plus radiotherapy 4.4 months, whereas combined treatment with surgery, radiation and chemotherapy had a median survival of 15 months [28]. Another retrospective review of 206 GBM patients ≥ 70, showed that on multivariate analysis, lack of surgical resection (HR 3.09; p < 0.001) was strongly independently associated with decreased OS [29], whilst a recursive partitioning analysis of prognostic factors in 437 glioblastoma patients ≥ 70 identified extent of surgery as the most important predictor of survival [19]. Tanaka et al retrospectively reviewed 105 patients ≥ 65 and showed that on multivariate analysis extent of resection significantly affected OS (p = 0.04). They demonstrated that patients undergoing biopsy had a complication rate of 30.8 % compared to 18.8 % in those undergoing resection [30]. Oszvald et al in a subgroup analysis of 146 patients > 65 who underwent resection or biopsy showed that age is not a prognostic factor in patients undergoing tumour resection but was in those undergoing biopsies only [31].

In a prospective study of 111 patients of GBM > 65, Fiorentino et al showed that extent of surgery had significant impact on median survival time (p = 0.009) [4], whilst Harris et al showed that EOR had a significant effect on OS in a prospective study of 108 patients > 75 (p = 0.001). Patients who underwent GTR had an OS of 12.0 months (95 % CI 10.0–14.6) as compared to 6.7 months (95 % CI 5.0–10.0) in patients who underwent STR or biopsy only [32].

In the only randomised study looking at the effect of EOR on survival, Vuorinen et al randomised 30 patients > 65 with radiologically suspected malignant glioma to receive either biopsy or resection. On final analysis only 23 patients had malignant glioma of which 10 were diagnosed by resection whereas 13 were diagnosed by biopsy. The authors showed that survival time was 2.8 times longer in patients who underwent craniotomy as compared to those who underwent biopsy (95 % CI 1.004–7.568, p = 0.049). However, there was no significant difference in the time of deterioration between the two treatment groups (p = 0.057). The authors concluded that although craniotomy and resection resulted in statistically significant improvement in OS compared to biopsy, there was no significant difference in time to deterioration [33].

A *meta*-analysis of 12,607 patients ≥ 60 showed that compared to biopsy, resection resulted in improved OS (p < 0.001), progression free survival (PFS) (p < 0.001), post-operative KPS (p < 0.001) and mortality (p = 0.002), whilst GTR resulted in improved OS (p < 0.001), PFS (p < 0.001), and post-operative KPS (p = 0.016) compared to STR with no significant difference in morbidity or mortality. The authors concluded that EOR was associated with improved OS, PFS and post-operative performance score [34]. Similarly, a SEER registry of 20,705 patients showed that for patients ≥ 75 GTR resulted in 2-month survival advantage as compared to STR, compared to a 3-month survival advantage for patients < 75 (p < 0.001) [35].

However, some other studies have failed to show a correlation between EOR and OS. In a retrospective study of 31 patients with GBM ≥ 70, Kimple et al did not observe any significant change in survival with EOR. Median survival for patients who went GTR, STR and biopsy was 26.0, 13.2 and 20.6 weeks respectively (log-rank test, p = 0.26) [36]. Similarly, Brandes et al studied 58 GBM patients ≥ 65 and showed that type of surgery had no significant effect on prognosis (log rank test; p = 0.21) [37].

Finally, an interesting recent study showed that resection of recurrent GBM in patients > 65 seems to offer significant improvement in survival without significant increase in complications [38].

In conclusion, most studies and *meta*-analysis have shown that GTR is associated with improved survival. However, the studies are generally retrospective leading to selection bias; elderly patients who are medically fit and have good PS are more likely to be offered aggressive surgery. Of course, unresected residual disease may be biologically different and more resistant in elderly patients. Nevertheless, in our opinion the decision to offer aggressive surgery should be personalised on a case-to-case basis considering co-morbidities, PS and patient‘s wishes rather than using cut off age alone as a guide to make treatment decisions. Table 1 summarises the surgical studies.

**Table 1:** Summary of main outcome regarding the effects of surgery on elderly patients newly diagnosed with glioblastoma.

Author	Study	Age	Sample size	Main outcome of study
Kelly 1994	Retrospective	>65	128	6.75 months survival
Martinez 2007	Retrospective	>65	76	Survival rate in 1 year was 9.6 % vs 59 % in younger
Stark 2007	Retrospective	>60	345	16 months survival after GTR, radiotherapy and reoperation
Iwamoto 2009	Retrospective	>65	394	Overall median survival 8.6 months
Chaichana 2011	Retrospective	>65	205	Resection resulted in 4.5 months survival vs biopsy alone which resulted in 3 months survival
Chaichana 2011	Retrospective	>65	129	Age < 75 vs age > 75 survival 8.7 vs 5.1 months respectively. However, age was relevant only when other preoperative factors (e.g. neurology deficits, tumour size, COPD) were considered
Ewelt 2011	Retrospective	>65	103	Overall survival after surgery alone (resection or biopsy) was 2.2 months, surgery plus radiation was 4.4 months, and surgery, radiation, and chemotherapy was 15 months
Scott 2011	Retrospective	>70	206	Surgery resulted in significant overall survival in those over 70
Scott 2012	Retrospective	>70	437	Extent of resection is the most important predictor of survival
Tanaka 2013	Retrospective	>65	105	Extent of resection significantly affects survival
Oszvald 2012	Prospective	>65	146	Complete surgical resection resulted in significant survival compared to partial resection
Fiorentino 2015	Prospective	>65	111	Extent of surgery significantly improved survival
Harris 2017	Prospective	>75	108	Extent of surgery was a significant factor for survival
Vuorinen 2003	Randomised trial	>65	30	Resection increased overall survival but not the time to deterioration compared to biopsy alone
Almenawer 2015	Meta-analysis	>60	12,607	Gross total resection increased survival compared to subtotal resection or biopsy alone
Noorbakhsh 2014	Retrospective	>75	20,705	Gross total resection increased survival compared to subtotal resection
Kimple 2010	Retrospective	>70	31	Extent of resection did *not* improve survival
Brandes 2009	Retrospective	>65	58	Type of surgery did *not* improve survival
Nunez 2020	Retrospective	>65	39	Resection for recurrent glioblastoma in the elderly offers significant survival benefit

### Radiotherapy

Villa et al investigated the effect of radiotherapy in 85 malignant glioma patients ≥ 65. Of these, 64 (75.3 %) were diagnosed with GBM, 32 (37.6 %) underwent biopsy, and mean KPS was 60. Median OS for all patients was 18.1 weeks whereas median survival of the 43 patients who started radiotherapy was 45.0 weeks. In multivariate analysis, radiotherapy was the only independent prognostic variable for survival [39]. Similarly, Mohan et al conducted a retrospective review of 102 GBM patients ≥ 70. 58 patients were treated with definitive radiotherapy, 19 received palliative radiotherapy whereas 25 patients did not receive radiation, with median survivals of 7.3, 4.5 and 1.2 months respectively (p < 0.0001) [18]. Whilst a retrospective review of 202 GBM patients ≥ 70 demonstrated a median survival of 10.6 months with radiotherapy compared to 1.9 months in patients who did not receive radiotherapy (p < 0.0001). In a multivariate analysis, radiotherapy was the only prognostic factor for survival (HR 8.9; p < 0.001) [40].

The only randomized trial included 85 patients with anaplastic astrocytoma or GBM ≥ 70 with KPS ≥ 70 and compared supportive care with radiotherapy (50 Gy in 28 fractions). The trial was discontinued as early analysis showed statistically significant survival improvement with radiotherapy. Analysis of 81 GBM patients (median age 73, median follow up 21 weeks) showed that for the 39 patients who received radiotherapy the median OS was 29.1 weeks compared with 16.9 weeks for patients who received supportive care with no difference in quality of life or cognition [41].

Kimple et al conducted a retrospective review of 31 GBM patients ≥ 70, demonstrating a median OS of 8.6 weeks for patients who received best supportive care compared to 28.2 weeks for patient treated with radiotherapy [36]. Similarly, in a SEER registry study of 2,836 GBM patients > 70, median survival of patients who received best supportive care, surgery only, radiotherapy only and surgery plus radiotherapy were 2.0, 3.0, 4.0 and 8.0 months respectively. Multivariate analysis showed that radiotherapy significantly improved cancer-specific survival (HR = 0.43; 95 % CI = 0.38–0.49) [11]. Whilst a further retrospective review of 206 GBM patients ≥ 70 demonstrated that on multivariate analysis, lack of radiotherapy carried a HR for death of 2.60 (95 % CI 1.57–4.31; p < 0.001) [29].

In conclusion, there is consistent evidence from several studies that the addition of radiotherapy improves survival, and this has led radiotherapy to be considered the standard of care for fit elderly patients. Table 2 summarises the radiotherapy studies.

**Table 2:** Summary of main outcomes of studies looking into the effects of radiotherapy in the management of elderly patients with GBM.

Author	Study	Age	Sample size	Main outcome of study
Villa 1998	Retrospective	>65	85	Radiotherapy increases survival. However, in patients older than 70 with malignant glioma the effects of RT is very limited
Mohan 1998	Retrospective	>70	102	In patients with good performance status RT significantly increases survival
Marijnen 2005	Retrospective	>70	202	RT significantly improves survival
Kimple 2010	Retrospective	>70	31	RT significantly improves survival compared to best supportive care
Scott 2011	Retrospective	>70	2836	RT significantly improves survival compared to best supportive care
Scott 2011	Retrospective	>70	206	Radiotherapy increased overall survival from 1.9 months to 6.7 months

## Radiotherapy schedules

The effective use of radiotherapy must balance tumour control with the risk of severe normal-tissue toxicity. Whilst the optimal dose of radiation for younger GBM patients is generally accepted, the side effects of radiotherapy on neuro-oncological patients is an ongoing debate [42]. One mechanism of radiotherapy toxicity relates to microvasculature disruption, and elderly people, with their higher incidence of diabetes and atherosclerosis, are at increased risk of radiation induced encephalopathy and cognitive decline [7], [43], [44]. Similarly, radiotherapy induced brain demyelination is significantly correlated with age, developing in 72.9 % of patients ≥ 50 compared to 39.2 % of patients < 50 [45]. Additionally, the risk of radiation induced neurotoxicity is dose-dependent [8], [43].

The standard 6-week radiotherapy schedule (60 Gy in 30 fractions) can also have significant impact on quality of life of elderly frail GBM patients as it requires daily transportation to the treatment centre. Therefore, several studies have investigated the effect of hypofractionated radiotherapy (HFRT) on median survival, toxicity and quality of life as it shortens treatment duration.

Bauman et al examined the effect of short course whole brain radiotherapy (30 Gy in 10 fractions) in 29 GBM patients ≥ 65 with KPS ≤ 50. Median survival was 6.0 months whereas median survival of similar patients in other studies treated radically or with supportive care were 10.0 and 1.0 month respectively. However, in the subset of patients with KPS > 50, radical radiotherapy was better than short course whole brain radiotherapy [46]. A further phase II study in 25 malignant glioma patients ≥ 70 with a median KPS of 70 investigated HFRT (37.5 Gy in 15 fractions). The median survival of the whole group was 8 months (95 % CI 4.8–9.6) whereas patients with KPS > 70 had a median survival of 10.4 months (95 % CI 9.6–14.7) [47]. Whilst a randomised trial of 68 patients comparing standard radiotherapy with HFRT (35 Gy in 10 fractions) showed a median survival of 10.3 months (95 % CI = 7.8–14.0 months) in the standard arm compared to 8.7 months (95 % CI = 7.4–10.7 months) with HFRT which was not statistically significant when adjusted for other variables [48].

Chang et al conducted a retrospective review of 59 GBM patients (median age 65) who received HFRT (50 Gy in 20 fractions) and showed that HFRT can be useful in some selected patients with GBM without significant decrease in survival or increase in toxicity [49].

Roa et al in a randomized trial assigned 100 GBM patients ≥ 60 to receive standard radiotherapy or HFRT (40 Gy in 15 fractions) post-surgery. Both groups were approximately matched for age, PS and type of surgery. Although the trial was closed early because of slow accrual, and hence was underpowered, it showed that OS was 5.6 months for HFRT compared to 5.1 months with standard radiotherapy (log-rank test, p = 0.57). The study also showed that of the patients completing radiotherapy, 49 % who received standard treatment required an increase in post-treatment corticosteroid compared to only 23 % only with HFRT (χ2 test, p = 0.02) [50].

Lutterbach and Ostertag conducted a retrospective review of 96 patients with GBM patients aged ≥ 60 years, who received either HFRT (42 Gy in 12 fractions) or standard radiotherapy demonstrating a median survival of 7.3 months with HFRT compared to 5.6 months with standard radiotherapy. 1 and 2-year survival was 60 % and 26 % with HFRT, compared with 49 % and 18 % with standard radiotherapy [51]. Donato et al compared the effect of standard radiotherapy to HFRT (30 Gy in 10 fractions). 22 patients received standard radiotherapy whereas 21 received HFRT. Median survival time in conventional and HFRT groups were 8.2 and 6.2 months respectively. 1-year OS in conventional and HFRT groups was 36 % and 23 % respectively [52]. Idbaih et al investigated the effect of HFRT (40 Gy in 15 fractions) in patients with GBM ≥ 70 with KPS ≥ 70 and showed that the median OS was 50.6 weeks (95 % CI = 26.3–62.0 weeks), and median PFS was 21.6 weeks (95 % CI = 17.0–39.9 weeks) in this group of patients [53].

Arvold et al conducted a retrospective review of 135 GBM patients ≥ 65 comparing HFRT (40 Gy in 15 fractions) versus standard radiotherapy with or without temozolomide. The study showed similar OS with HFRT and standard radiotherapy with concurrent temozolomide (HR = 1.10; 95 % CI = 0.50–2.4, p = 0.82). Though there was selection bias in the study as patients who received HFRT were significantly older and of poor PS which the authors controlled with cox regression and propensity score analyses [54].

Roa et al conducted a randomised phase III trial in 98 patients with newly diagnosed glioblastoma (≥65 or ≥ 50 with KPS 50–70) and compared HFRT (40 Gy in 15 fractions) to palliative radiotherapy (25 Gy in 5 fractions). The authors showed that the median OS with palliative radiotherapy was 7.9 months (95 % CI 6.3–9.6 months) compared to 6.4 months (95 % CI 5.1–7.6 months) with HFRT (p = 0.988). With a median follow-up time of 6.3 months, the quality of life between both groups at 4 and 8 weeks after treatment was also not different. However, as the follow up was limited, late neurotoxicity of palliative radiotherapy could not be evaluated [55]. A subset analysis of patients ≥ 65 showed that median OS was 6.8 months in patients who received palliative radiotherapy (95 % CI 4.5–9.1 months) compared to 6.2 months (95 % CI 4.7–7.7 months) in patients who received HFRT (p = 0.936). The median PFS time was 4.3 months (95 % CI 2.6–5.9 months) and 3.2 months (95 % CI 0.1–6.3 months) with palliative radiotherapy and HFRT respectively (p = 0.706) [56].

However, some other studies have shown conflicting results. A Medical Research Council randomized trial in 474 patients with malignant glioma aged 18–70 (30 % >60) compared standard radiotherapy with HFRT (45 Gy in 20 fractions) and showed that there was a statistically significant prolongation of median survival from 9 months with HFRT to 12 months with standard radiotherapy (HR = 0.75, χ2 = 7.36, p = 0.007), whilst short term morbidity was equivalent [57]. However, they did not compare long-term toxicities and did not include patients > 70.

Fiorentino et al analysed 111 GBM patients > 65 (KPS > 70; Charlson Comorbidity Index < 3) from 4 prospective phase II trials and demonstrated on multivariate analysis that higher radiation dose significantly impact OS (p = 0.02; HR = 0.3; 95 % CI = 0.10–0.87). Though the authors acknowledge that the result in their study may be influenced by small sample size and inclusion criteria [4].

In conclusion, though standard radiotherapy and HFRT have been shown to improve OS in elderly patients with GBM, the most appropriate dose of radiotherapy has not been established conclusively. More research is needed to compare the effect of standard radiotherapy and HFRT on quality of life and long-term radiotherapy induced neurotoxicity, and to identify factors that predict for the development of radiotherapy toxicity to allow personalisation of radiotherapy dose decisions.

### Chemotherapy

Following concerns regarding the neurotoxicity risk from radiotherapy, alongside the fact subgroup analysis from the Stupp study^2^ showing reduced benefit from chemoradiotherapy in elderly populations, several studies have investigated the efficacy of chemotherapy compared to radiotherapy as monotherapy. Temozolomide monotherapy in elderly also appears attractive as the incidence of O(6)-methylguanine-DNA methyltransferase (MGMT) promoter methylation in elderly population is comparable to younger patients [58], [59].

Glantz et al conducted a retrospective review of 86 patients with malignant glioma > 70 treated with standard radiotherapy or temozolomide (150–200 mg/m^2^/day, 5 days every 28 days). 54 patients (63 %) received radiotherapy whereas 32 patients (37 %) received temozolomide. Median OS with temozolomide and radiotherapy were 6.0 months and 4.1 months respectively (log rank test, p = 0.198), whilst 1-year survival rates were 11.9 % and 9.3 % respectively. The study demonstrated that more patients receiving temozolomide died at home compared to those receiving radiotherapy though this was not statistically significant (p = 0.423, chi-square analysis). However, this study did not assess MGMT promoter methylation status, one of the main predictors of response to temozolomide [7].

Chinot et al in a phase II study looked at the effect of primary temozolomide (150–200 mg/m^2^/day, 5 days every 28 days) in patients > 70. None of the patients received radiotherapy. The authors identified 9 patients (31 %) with partial response, 12 patients (41 %) with stable disease whereas 8 patients (28 %) disease progressed. Temozolomide monotherapy was safe and well tolerated with grade 3/4 thrombocytopenia and neutropenia seen in 6 % and 9 % of patients respectively [60].

Chamberlain et al studied 15 patients ≥ 70 (KPS ≥ 50) treated with adjuvant temozolomide and deferred radiotherapy following surgery. Temozolomide (75 mg/m^2^/day) was given as 42 days on and 14 days off regimen up to 3 cycles with median OS of 6.0 months (range 4.0–14.0 months) and acceptable toxicity [61].

Laigle-Donadey et al conducted a retrospective review of 39 GBM patients treated with temozolomide alone following radiotherapy refusal. Median age was 75 (range 70–83) and median KPS was 70 (range 70–80). Median OS was 36 weeks for the whole group. Complete response was seen in one patient with 10 partial responses. Grade 3/4 toxicity occurred in 8 patients [62].

Scott et al conducted a retrospective review of 206 patients and found that patients receiving chemotherapy had a median survival of 11.2 months as compared to 3.5 months for patients not receiving chemotherapy (p < 0.001). On multivariate analysis, not receiving chemotherapy carried HR for death of 2.20 (p < 0.001) [29].

In a nonrandomised phase II trial, ANOCEF group investigated the effect of temozolomide (150–200 mg/m^2^/day, 5 days every 28 days) in patients ≥ 70 with KPS < 70. None of the patients received radiotherapy and < 10 % patients received second-line chemotherapy. The median OS was 25.0 weeks (95 % CI 19.0–28.0 weeks) which compared favourably with 12.0–16.0 weeks median OS with historic controls. Median OS in patients with MGMT promoter methylation was 31.0 weeks (95 % CI 25.0–46.8 weeks) compared to 18.7 weeks (95 %CI 8.5–26.2 weeks; p = 0.03) in patients with unmethylated MGMT promoter. Surprisingly in 23 patients (33 %) KPS improved by 10 or more points and 18 patients (26 %) became self-caring (KPS ≥ 70). Authors also noted that quality of life and cognition improved with temozolomide prior to disease progression. Grade 3/4 neutropenia and thrombocytopenia was seen in 13 % and 14 % of patients respectively [63].

In an international randomised phase III study (NORDIC Study), newly diagnosed GBM patients > 60 were recruited from 7 countries and allocated to either 200 mg/m^2^ temozolomide for 5 days in a 28-day cycle for a total of 6 cycles, HFRT (34 Gy in 10 fractions) or standard radiotherapy. A total of 342 patients were recruited. 291 were randomised into 3 groups (standard radiotherapy = 100, temozolomide = 93, HFRT = 98) whereas 51 patients were randomised into two groups only (temozolomide = 26, HFRT = 25) as some centres did not offer standard radiotherapy. Median OS with standard radiotherapy, temozolomide and HFRT was 6.0 months (95 % CI 5·1-6·8), 8·3 months (95 % CI 7·1-9·5, HR 0.7p = 0.01) and 7·5 months (95 % CI 6·5-8·6, HR 0.85p = 0.24) respectively. Subgroup analysis showed that patients with MGMT promoter methylation had a median OS of 9·7 months (95 % CI 8·0-11·4) compared to 6·8 months in unmethylated MGMT patients (95 % CI 5·9-7·7; HR 0·56, p = 0·02). However, in patients who received radiotherapy, MGMT promoter methylation had no effect on OS (HR 0·97; p = 0·81). One of the drawbacks of this study was the fact that both patient and clinician were not blinded to the treatment received [64].

In another phase III trial (NOA-08 Study), patients > 65 and KPS ≥ 60 were randomised to temozolomide (100 mg/m^2^, one week on and one week off) or standard radiotherapy. In 373 patients analysed, median OS with temozolomide was 8·6 months (95 % CI 7·3-10·2) compared to 9·6 months (8·2-10·8) with radiotherapy. Patients with MGMT methylation had a median OS of 11.9 months [95 % CI 9·0 - to not reached] compared to 8.2 months (95 % CI 7·0-10·0) in patients without MGMT promoter methylation (HR 0·62, p = 0·014). However, the temozolomide group had a higher incidence of neutropenia, thrombocytopenia, lymphocytopenia, infection, thromboembolism, and deranged liver enzyme levels [65].

In a *meta*-analysis comparing temozolomide versus radiotherapy in newly diagnosed patients with GBM ≥ 65, only 2 randomized clinical trials and 3 comparative studies were included in the final analysis. The analysis showed an OS advantage with temozolomide compared to radiotherapy (HR 0.86, 95 % CI 0.74–1.00). However, a sensitivity analysis of 2 randomized clinical trials showed that temozolomide is only non-inferior to radiotherapy (HR 0.91, 95 % CI 0.66–1.27). Among patients receiving temozolomide, MGMT promoter methylation group had a longer OS compared to those with non-methylated MGMT promoter (HR 0.50, 95 % CI 0.35–0.70). Authors also showed an interaction with MGMT promoter methylations status, such that in patients with MGMT promoter methylation, temozolomide resulted in improved OS compared to radiotherapy (HR 0.66, 95 % CI 0.47–0.93) whereas the opposite was true for patients with non-methylated MGMT (HR 1.32, 95 % CI 1.00–1.76). Quality of life was equivalent in both groups [66].

Additionally, some studies, have found no interaction between MGMT promoter methylation and OS [67]. There remains outstanding questions as to the specific use of MGMT promoter status as a biomarker. Whilst theoretically it would be expected to act as a predictive biomarker for response to temozolomide, the greater evidence is for its role as a general prognostic indicator. Additionally, given the variety of methods currently in use for assessing MGMT promoter methylation, which often give disparate results, the optimal method and cut off for classifying tumours as MGMT promoter methylated or unmethylated remains unknown [68].

However, response to chemotherapy and chemotherapy toxicity is strongly related to age. A retrospective review of 148 patients with malignant astrocytomas or recurrent astrocytomas, showed that median survival in patients < 60 after nitrosourea based chemotherapy was 43 weeks compared to 24 weeks in patients ≥ 60 (p < 0.001), and the risk of myelosuppression requiring hospitalization was significantly lower in patients < 60 compared to patients ≥ 60 (p = 0.03) [69].

In conclusion, though phase III randomised studies have shown improved survival in elderly patients with MGMT promoter methylation treated with temozolomide, at present there is insufficient evidence to recommend temozolomide as a monotherapy in elderly GBM patients who are fit for multimodality treatment as there have been no randomised trials comparing standard chemoradiotherapy to temozolomide monotherapy alone. However, for patients unfit for combination oncological treatment and with MGMT promoter methylation, then single agent temozolomide can be expected to provide equivalent survival benefit to radiotherapy, but without the significant risks of cognitive impairment and other radiation toxicities. Table 3 summarises the chemotherapy studies.

**Table 3:** Summary of the effects of chemotherapy in elderly GBM patients.

Author	Study	Age	Sample size	Main outcome of study
Glantz 2003	Retrospective	>70	86	Chemotherapy is as effective as radiotherapy
Chinot 2004	Prospective	>70	32	Chemotherapy (temozolomide) is safe and effective in elderly patients with GBM
Gállego Pérez-Larraya 2011	Prospective nonrandomised trial	>70	70	Chemotherapy increased overall survival to 25 weeks compared to best supportive care 12–16 weeks
Malmstorm 2012	Randomised trial	>70	342	Temozolomide (and hypofractionated radiotherapy) is superior to standard radiotherapy in improving overall survival
Wick 2012	Randomised trial	>65	373	Temozolomide not inferior to radiotherapy
Yin 2014	Meta-analysis	>65	993	Temozolomide is more beneficial than radiotherapy for overall survival

### Chemoradiotherapy

Prior to the Stupp^2^ era, a *meta*-analysis of 12 randomized trials comprising > 3,000 patients compared chemoradiotherapy with radiotherapy alone demonstrating a 15 % relative decrease in risk of death with chemoradiotherapy (p < 0.0001). Though not specifically looking at the elderly GBM population, the benefit of chemoradiotherapy was independent of age, sex, extent of resection, or PS [70]. Similarly, Brandes et al conducted a prospective nonrandomised trial in 79 GBM patients > 65, in which 24 patients received radiotherapy alone, 32 patients received radiotherapy plus procarbazine, lomustine and vincristine (PCV) and 22 received radiotherapy and temozolomide. Better OS with radiotherapy plus temozolomide (14.9 months) was seen compared to radiotherapy alone (11.2 months; p = 0.002) but not radiotherapy plus PCV (12.7 months), although grade III/IV toxicity was higher with PCV compared to temozolomide [71].

The Stupp protocol^2^ was evaluated in a prospective trial of 32 GBM patients **≥** 70 with KPS **≥** 70 and identified a median OS of 10.6 months and 6-month and 12-month survival was 91 % and 37 % respectively. Grade 3–4 haematological toxicity occurred in 28 % of patients whereas neurotoxicity occurred in 40 %, most of which resolved with steroids [72].

In a retrospective review of 394 GBM patients **≥** 65, Iwamoto et al showed that chemoradiotherapy was associated with a 55 % reduction in the risk of death (HR = 0.45; 95 % CI 0.30–0.66; p < 0.0001]) after adjusting for age, PS, extent of resection, and number of lesions [25]. Similarly, in a retrospective review of 31 patients with GBM **≥** 70, Kimple et al showed the median survival for patients who received best supportive care, radiotherapy alone and chemoradiotherapy were 8.4, 28.2 and 50.5 weeks respectively (log rank test, p < 0.0001). Of 13 patients who received chemoradiotherapy, toxicity data was available for 10, of whom 3 patients (30 %) had treatment related toxicity [36].

Several other studies have shown that chemoradiotherapy is associated with improved OS. In a retrospective review of 291 GBM patients ≥ 65, Barker et al showed that chemoradiotherapy was associated with significantly improved survival (p < 0.01). In patients with favourable prognostic factors, chemoradiotherapy improved median survival and 2-year OS in patients 65–70 from 12.0 months to 21.0 months and from 14 to 41 % respectively. Also, in patients **≥** 71, chemoradiotherapy improved median survival and 2-year OS from 10.0 to 13.0 months and from 5 to 24 % [73]. In a retrospective review of 105 patients **≥** 65 by Tanaka et al, follow up data was available for 84 patients and showed that with chemoradiotherapy the median PFS and OS were 8.0 and 12.5 months respectively [30]. Similarly, a combined analysis of 111 patients > 65 from 4 prospective trials assessed the safety and efficacy of chemoradiotherapy. The median OS was 13 months whereas 2, 3 and 5-year survival rates were 28.6 %, 16.2 % and 2 % respectively. 19.82 % of the patients experienced neurological symptoms during chemoradiotherapy [4].

In another retrospective review of 74 patients **≥** 65, Putz et al showed that on univariate analysis, for a cumulative dose of concurrent temozolomide > 2655 mg/m^2^ the median survival was 13.9 months compared to 4.9 months with cumulative dose ≤ 2655 mg/m^2^ (p = 0.0216). Multivariate analysis confirmed that cumulative dose of concurrent temozolomide > 2655 mg/m^2^ was a significant independent prognostic factor (HR = 0.33; p = 0.002) Interestingly, in subgroup analysis, in patients who received > 2655 mg/m^2^ of concurrent temozolomide, adjuvant temozolomide did not statistically significantly improve survival (p = 0.145) [74]. In a further retrospective review of 14,866 GBM patients ≥ 70 years from the National Cancer Data Base (2004–2012), Amsbaugh et al showed that percentage of patients who received best supportive care, radiotherapy alone, chemotherapy alone and chemoradiotherapy was 26.6 %, 13.9 %, 4.4 % and 55.2 % respectively. The median OS was 3.42 months (95 % CI 3.25–3.52 months; p < 0.0001), 5.29 months (95 % CI 4.99–5.52 months), 4.67 months (95 % CI 4.30–5.16 months) and 9.20 months (95 % CI 8.97–9.46 months) respectively [75].

In a *meta*-analysis of 16 non-randomised studies comprising 1,492 patients, Yin et al showed that chemoradiotherapy was associated with decreased risk of death compared to radiotherapy alone in elderly GBM patients (HR = 0.59, 95 % CI 0.48–0.72). However, the chemoradiotherapy was associated with increased haematological toxicity [76].

However, the major drawbacks of most of these studies was the retrospective nature of the study along with lack of information regarding PS, toxicity, MGMT promotor methylation status and quality of life data.

To investigate chemoradiotherapy toxicity, Sijben et al conducted a retrospective review of 39 GBM patients **≥** 65. 19 patients received chemoradiotherapy whereas 20 received radiotherapy alone. The median OS in chemoradiotherapy and radiotherapy group was 8.5 months (range 2.0–24.7 months) and 5.2 months (range 1.5–14.2 months) respectively which the authors thought could be due to differences in extent of resection, age, and PS between the two groups. Also 42 % of the patients who received chemoradiotherapy had grade 3/4 treatment related toxicity as compared to none who received radiotherapy alone [68]. Saito et al compared toxicity with chemoradiotherapy in patients **≥** 65 vs < 65 years and identified grade 4 toxicity of 26 % and 8 % respectively (p = 0.046) [77]. Brandes et al studied the effect of chemoradiotherapy in 58 patients ≥ 65 with KPS ≥ 70 and showed that grade 3/4 toxicity during concomitant treatment occurred in 11/58 patients (19 %) whereas it was seen in 24/48 patients (50 %) during adjuvant treatment [37].

To reduce chemoradiotherapy toxicity, several groups have investigated the efficacy of low dose radiotherapy and/or chemotherapy on OS in patients with GBM.

In a single arm study, Minniti et al investigated the effect of HFRT (30 Gy/6 fractions over 2 weeks) followed by up to 12 cycles of adjuvant temozolomide (150–200 mg/m^2^ for 5 days during 28-day cycle) in 43 GBM patients ≥ 70 with KPS ≥ 60. The study showed that the median OS was 9.3 months whereas 6 and 12-month survival rates were 86 % and 35 % respectively. Though grade 3/4 hematologic toxicity was seen in 28 % of patients during adjuvant treatment, there was no significant adverse effect on quality of life [78].

Investigating the effect of low dose temozolomide with concurrent radiotherapy, Combs et al studied 43 GBM patients **≥** 65. Patient received standard radiotherapy along with either 50 mg/m^2^ temozolomide (38 patients) or 75 mg/m^2^ temozolomide (8 patients). The authors showed that the median OS was 11 months whereas 1 and 2-year survival was 48 % and 8 % respectively [79].

Minniti et al in a phase II multicentre trial investigated the effect of HFRT (40 Gy in 15 fractions) with concomitant temozolomide (75 mg/m^2^) followed by 12 cycles of adjuvant temozolomide (150–200 mg/m^2^ for 5 days during 28-day cycle) in patients ≥ 70 and KPS ≥ 60. Median OS was 12.4 months whereas 1 and 2-year survival rates was 58 % and 20 % respectively. However, 22 % of patients experienced grade 3/4 treatment related toxicity [80].

In another retrospective study of 243 GBM patients ≥ 65, Minniti et al investigated the effect of standard chemoradiotherapy or HFRT (40 Gy in 15 fractions) with concomitant and adjuvant temozolomide. 127 patients received standard chemoradiotherapy whereas 116 patients received HFRT plus chemotherapy. Median OS was 12.0 and 12.5 months respectively. Standard chemoradiotherapy as compared to HFRT plus chemotherapy was associated with increased toxicity, use of steroids, and deterioration in PS during treatment [81].

In their landmark paper, Perry et al conducted a randomised trial to compare HFRT (40 Gy in 15 fractions) alone to HFRT with concomitant (75 mg/m^2^ for 21 days) and 12 cycles adjuvant temozolomide (150–200 mg/m^2^/day, 5 days every 28 days). 281 GBM patients **≥** 65 were randomised to each arm. Median OS with chemoradiotherapy and radiotherapy was 9.3 months and 7.6 months respectively (HR = 0.67; 95 % CI 0.56–0.80; p < 0.001). The median PFS in both groups was 5.3 months and 3.9 months respectively (HR = 0.50; 95 % CI 0.41–0.60; p < 0.001). In 165 patients with MGMT promoter methylation, median OS in chemoradiotherapy and radiotherapy alone group was 13.5 months and 7.7 months respectively (HR = 0.53; 95 % CI 0.38–0.73; p < 0.001). There was no significant difference in quality of life between the two groups [15]. This was a well design trial which tried to overcome most of the shortcomings of previous studies investigating the effect of chemoradiotherapy on elderly GBM patients; it was a prospective randomised study which included data on MGMT promoter methylation status as well as quality of life measures.

However, this trial raised several outstanding questions. To be eligible for this trial, patients had to be deemed unsuitable for standard radiotherapy, meaning that the study has not answered the question about the best treatment modality for elderly patients with GBM who are fit to receive standard radiotherapy. Also, in a way the study assumes that HFRT is inferior to standard radiotherapy as HFRT was offered as second option to the patients. To be eligible for this study, patients needed to have Eastern Cooperative Oncology Group PS 0–2, but still be ineligible for standard chemoradiotherapy. It is therefore unclear what the ideal treatment for elderly GBM patients with poor PS (≥3). Interestingly the study also showed that in 189 patients with unmethylated MGMT, the median OS was 10.0 months with chemoradiotherapy as compared to 7.9 months with radiotherapy alone (HR = 0.75; 95 % CI 0.56–1.01; p = 0.055). Whilst this difference was not statistically significant the fact that temozolomide can be effective in patients with nonmethylated MGMT status is intriguing and raises the question of should we be treating all patients of GBM with temozolomide irrespective of their MGMT methylation status.

Subgroup analysis of this trial also showed that the median OS was better in older patients compared to younger patients with a median OS in patients 65–70 and ≥ 76 of 8.7 and 10.0 months respectively. The authors of the study acknowledge that this may be due to selection bias in the study with younger elderly patients who were too frail to receive standard radiotherapy were chosen for this study along with fitter more elderly GBM patients. It is also difficult to interpret the results of this study in view of the findings of other trials. Patients treated with chemoradiotherapy in this study had survival comparable to other studies where patients have only received radiotherapy or temozolomide as monotherapy; so the role of radiotherapy or temozolomide as monotherapy in management of GBM in elderly is still unclear.

More recently, Navarria et al evaluated the effects of HFRT in 30 elderly GBM patients (mean age 75, all > 70) and concurrent and/or adjuvant temozolomide [82]. 43 % of the patients underwent resection and 57 % biopsy. This showed that in selected elderly patients HFRT treatment is a reasonable option with an acceptable complication profile and no need to increase steroid usage [82]. Similarly, another retrospective study investigating 30 elderly (>75) GBM patients treated with HFRT and chemotherapy either with temozolomide alone (n = 20) or temozolomide and bevacizumab (n = 10), identified OS and PFS of 12.9 and 9.9 months respectively with an acceptable toxicity profile. Interestingly, MGMT status was not a significant factor affecting survival [83]. Both studies, were single arm trials without a control group which makes the interpretation of the results more cautious.

A recent systematic review assessed the survival outcome of HFRT plus temozolomide versus standard chemoradiotherapy in elderly patients diagnosed with GBM and concluded that in well selected elderly patients with GBM the combination of HFRT and temozolomide seems to offer a similar PFS to standard chemoradiotherapy [84].

A very recent retrospective study of 128 GBM patients > 70 (15 % >80, majority PS < 2) investigated standard chemoradiotherapy following either complete/partial surgical resection or biopsy. 81 % of patients completed treatment with a median OS and PFS of 11.7 and 9.5 months respectively. Survival and toxicity profile of this group of patients was not worse their younger counterparts and, interestingly, post-operative neurological impairment and age > 80 was not linked to a worsened outcome. However, limitations of the study were the lack of information regarding MGMT status and its retrospective nature [85].

Attempting to use a less toxic regime to conventional chemotherapy that would still potentiate and enhance the effects of short course RT in elderly patients, Brazil et al. assessed the addition of hydroxychloroquine to short course radiotherapy (30 Gy in 10 fractions) in patients > 70 with newly diagnosed GBM in a phase II trial. Unfortunately, the results did not show any survival benefit compared to radiotherapy alone [86]. Nevertheless, there are ongoing attempts to discover alternative ways of enhancing the effects of radiotherapy in elderly GBM patients for whom standard treatment may not be appropriate or tolerated including the PARADIGM trial assessing Olaparib as a radiosensitizer in patients > 65 which completed recruitment in June 2022 with the results expected imminently (https://www.cancerresearchuk.org/about-cancer/find-a-clinical-trial/a-trial-looking-at-olaparib-and-radiotherapy-for-people-with-glioblastoma-paradigm).

For those patients who are deemed fit, then the addition of chemotherapy to radiotherapy has been shown to consistently improve survival compared to radiotherapy alone. However, the optimal radiotherapy regime is still undecided, with some suggestion that HFRT is at least comparable to conventional fractionation in terms of survival and is possibly better tolerated. Research is ongoing to develop novel combinations for patients who are unsuitable for temozolomide chemotherapy and in patients with an unmethylated MGMT promoter who derive less benefit from temozolomide. Table 4 summarises the studies of chemoradiotherapy.

**Table 4:** Summary of the effects of chemoradiotherapy in newly diagnosed elderly GBM patients.

Author	Study	Age	Sample size	Main outcome of study
Minniti 2008	Prospective	>70	32	Survival of elderly GBM patients on Stupp protocol: 10.6 months
Iwamoto 2009	Retrospective	>65	394	Chemoradiotherapy reduced risk of death in 55 % of patients
Kimple 2010	Retrospective	>70	31	Median survival following best supportive care, radiotherapy alone and chemoradiotherapy is 8.4, 28.2 and 50.5 weeks respectively
Barker 2012	Retrospective	>65	291	In those > 71 chemoradiotherapy improved median survival and 2-year overall survival from 10.0 to 13.0 months and from 5 to 24 % respectively
Tanaka 2013	Retrospective	>65	105	Chemoradiotherapy increased the median PFS and OS to 8.0 and 12.5 months respectively
Fiorentino 2015	Meta-analysis	>65	111	Median overall survival was 13 months whereas 2, 3 and 5-year survival rate was 28.6 %, 16.2 % and 2 % respectively
Amsbaugh 2017	Retrospective	>70	14,866	Median overall survival for patients who received best supportive care, radiotherapy alone, chemotherapy alone and chemoradiotherapy was 3.42, 5.29, 4.67 and 9.20 months respectively
Yin 2013	Meta-analysis	>65	1096	Chemoradiotherapy was associated with decreased risk of death compared to radiotherapy alone
Sijben 2008	Retrospective	>65	39	Median overall survival in chemoradiotherapy and radiotherapy group was 8.5 and 5.2 months
Combs 2008	Retrospective	>65	43	Radiotherapy and temozolomide resulted in median overall survival of 11 months whereas 1 and 2-year survival was 48 % and 8 % respectively
Minniti 2015	Retrospective	>65	243	Median survival with standard radiotherapy was 12 months whereas survival with short-term radiotherapy and temozolomide was 12.5 months
Perry 2017	Randomised trial	>65	562	Chemoradiotherapy (temozolomide and short-course radiotherapy) increased survival to 9.3 months compared to radiotherapy alone 7.6 months
Navarria 2018	Prospective	>70	30	Hypofractionated radiation treatment in elderly glioblastoma patients is a reasonable treatment with limited morbidity
Ohno 2019	Retrospective	>75	30	Hypofractionated radiation treatment in elderly glioblastoma patients is a reasonable treatment option irrespective of MGMT status
Lu 2019	Systematic review	>65	917	Hypofractionated RT and temozolomide seems to have a similar effect to progression free survival as standard radiotherapy combined with temozolomide
Brazil 2020	Prospective	>70	54	Hydroxychloroquine combined with short course of radiotherapy in newly diagnosed elderly GBM patients did not improve survival.
Vaugier 2021	Retrospective	>70	128	81 % completed the entire 6-week Stupp chemoradiation protocol. Median overall survival was 11.7 months and median progression-free survival was 9.5 months.

### Recurrent disease

The management of recurrent disease is challenging for all patients with glioblastoma, with additional challenges in the elderly population due to the factors discussed previously and the greater heterogeneity of first line treatments. This topic has been reviewed in depth recently [87] and a full discussion is beyond the scope of this article.

The most accepted treatments for recurrent disease in the elderly include second resection, re-irradiation, and systemic therapy. Consistently, post-recurrence PS is the most important factor for deciding optimal treatment with some evidence that age is not an independent prognostic factor [88]. Additionally, receipt of any treatment has been associated with improved survival even in those with poor PS [89]. The additional benefits of re-irradiation in addition to systemic therapy remains contested the results of the phase 2 BRIOChe [90] and phase 3 LEGATO [91] trials are awaited. Both studies include recurrent GBM patients of any age with no upper age limit but require a KPS of 70 + or PS 0–2 respectively. Therefore, the appropriate tailoring of treatment to the individual patients is of key importance for optimising outcomes at recurrence.

## Conclusions

In conclusion, contrary to the standardised protocol for younger GBM patients, and although the landmark randomised trial by Perry et al [15] has proved transformative for clinicians formulating treatment strategies for elderly patients with GBM, in our opinion the optimal treatment of GBM in the elderly population is yet to be defined. Management of these patients remains challenging and the ability of the clinician to accurately assess the functional status of the patient, consider the wishes of the patient and the family, and bring all those variables into a neuro-oncology MDT seems the best way forward.

PS seems the most important determinant in deciding the best management strategy for elderly patients with newly diagnosed glioblastoma. It is therefore dependent on the clinician to identify those patients who are fit enough to undergo more intensive treatment. To improve the identification of the “fit” elderly patient, several tools have been developed and investigated, including the Comprehensive Geriatric Assessment [92], and these tools are now recommended by the International Society of Geriatric Oncology [93] and in several international guidelines [94]. Specifically for glioblastoma, the GOLDEN study [95] prospectively investigated a modified geriatric assessment tool in a multicentre feasibility study. They identified that completing a geriatric assessment is feasible and acceptable with patients, as well as identifying that baseline cognitive impairment, functional impairment and mobility impairment were all statistically significantly associated with worse survival outcomes.

**Fig. 1:**
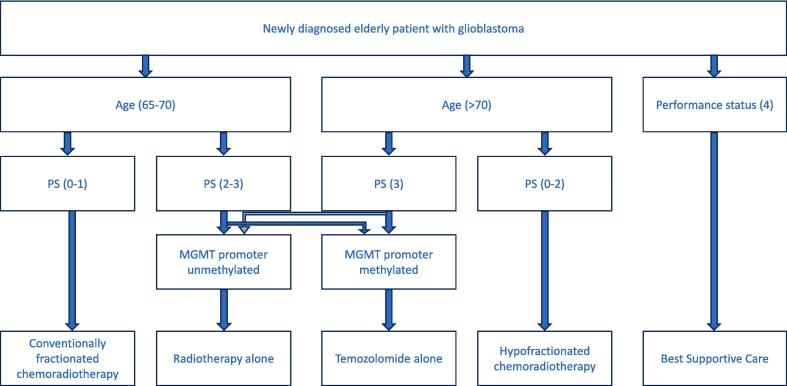
Flowchart of treatment recommendations for newly diagnosed elderly patients with glioblastoma, incorporating age, performance status and MGMT promoter methylation status.

In this context, elderly patients 65–70 with an excellent PS could be considered as candidates for standard treatment of surgery, standard radiotherapy with concomitant and adjuvant temozolomide (Fig. 1). Similarly, elderly patients > 70 with good PS could receive the above with HFRT instead of standard fractionation. In elderly GBM patients with poorer PS and MGMT promoter methylation temozolomide chemotherapy can be considered. There is no evidence to suggest an advantage of temozolomide in unmethylated patients. For elderly patients who cannot tolerate chemotherapy, HFRT can provide effective symptom palliation and improve survival [96].

### Open questions

Treatment of elderly GBM patients remains extremely challenging and despite ongoing research there are still important unresolved issues. Firstly, whether the biological behaviour of glioblastoma in the elderly is different to younger patients is not understood. This is a challenge for researchers to understand as any potential treatment will depend on better understanding of the genetic, molecular, and cellular mechanism of this disease. It might be that conventional treatment in the younger might not be as effective in the elderly simply because the biology is different. Despite the constant progress in understanding GBM [97], [98] we still do not fully understand the complexity of this disease. Unless we have a deeper understanding of the underlying biology, treatment regimes will have limited success. An improved understanding of the interactions between frailty, comorbidity and treatment is also necessary to facilitate effective treatment personalisation. Another, more general, challenge is how do we define elderly in the context of glioblastoma and how different stages of older age respond (or not) to treatment. As the population becomes older, it is becoming more and more important to answer these questions and develop age-specific treatment protocols and attract more funding to support basic and clinical research in glioblastoma in the elderly.

## Author contributions

1 guarantor of integrity of the entire study: **Nektarios K. Mazarakis, Stephen D. Robinson, Georgios Giamas.** 2 study concepts and design: **Nektarios K. Mazarakis**. 3 literature research: **Nektarios K. Mazarakis, Stephen D. Robinson, Priyank Sinha**. 4 clinical studies: N/A. 5 experimental studies/data analysis: N/A. 6 statistical analysis: N/A. 7 manuscript preparation: **Nektarios K. Mazarakis, Stephen D. Robinson, Priyank Sinha**. 8 manuscript editing: **Nektarios K. Mazarakis, Stephen D. Robinson, Priyank Sinha, Christos Koutsarnakis, Spyridon Komaitis, George Stranjalis, Susan C. Short, Paul Chumas, Georgios Giamas**

## Funding

This research did not receive any specific grant from funding agencies in the public, commercial, or not-for-profit sectors.

## CRediT authorship contribution statement

**Nektarios K. Mazarakis:** Writing-original draft. **Priyank Sinha:** Writing-original draft, Conseptualisation. **Stephen D. Robinson:** Writing-review & editing. **Christos Koutsarnakis:** Writing-Review & Editing. **Spyridon Komaitis:** Writing-Review & Editing. **George Stranjalis:** Supervision. **Susan C. Short:** Supervision, Writing-review & Editing. **Paul Chumas:** Supervision, Conceptualization. **Georgios Giamas:** Supervision, Writing-review and editing, Project administration, Funding acquisition.

## Declaration of competing interest

The authors declare that they have no known competing financial interests or personal relationships that could have appeared to influence the work reported in this paper.
